# Insights into the Role of Ketoreductases in the Biosynthesis of Partially Reduced Bacterial Aromatic Polyketides*

**DOI:** 10.1002/cbic.201900357

**Published:** 2019-12-09

**Authors:** Syed Masood Husain, Andreas Präg, Anton Linnenbrink, Andreas Bechthold, Michael Müller

**Affiliations:** ^1^ Centre of Biomedical Research SGPGIMS Campus Raebareli Road Lucknow 226014 Uttar Pradesh India; ^2^ Institut für Pharmazeutische Wissenschaften Albert-Ludwigs-Universität Freiburg Albertstrasse 25 79104 Freiburg Germany; ^3^ Institut für Pharmazeutische Wissenschaften Albert-Ludwigs-Universität Freiburg Stefan-Meier-Strasse 19 79104 Freiburg Germany

**Keywords:** asymmetric synthesis, enzyme catalysis, gene annotation, ketoreductases, polyketide biosynthesis

## Abstract

Partially reduced aromatic polyketides are bioactive secondary metabolites or intermediates in the biosynthesis of deoxygenated aromatics. For the antibiotic GTRI‐02 (mensalone) in different *Streptomyces* spp., biosynthesis involving the reduction of a fully aromatized acetyltrihydroxynaphthalene by a naphthol reductase has been proposed and shown in vitro with a fungal enzyme. However, more recently, GTRI‐02 has been identified as a product of the ActIII biosynthetic gene cluster from *Streptomyces coelicolor* A3(2), for which the reduction of a linear polyketide precursor by ActIII ketoreductase, prior to cyclization and aromatization, has been suggested. We have examined three different ketoreductases from bacterial producer strains of GTRI‐02 for their ability to reduce mono‐, bi‐, and tricyclic aromatic substrates. The enzymes reduced 1‐ and 2‐tetralone but not other aromatic substrates. This strongly suggests a reduction of a cyclized but not yet aromatic polyketide intermediate in the biosynthesis of GTRI‐02. Implications of the results for the biosynthesis of other secondary polyketidic metabolites are discussed.

## Graphical abstract

**One ring to rule**: In contrast to the case of reduction of fully aromatic naphthols or a linear polyketide, monocyclized polyketides are proposed as the substrates of ketoreductases in the biosynthesis of bacterial aromatic polyketides such as GTRI‐02.

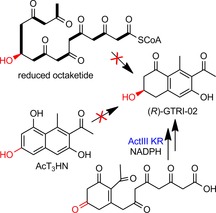

Aromatic polyketides, often characterized by the presence of polycyclic structures, represent a class of widely distributed secondary metabolites.^1^, ^2^, ^3^ Many of these polyketides are used as drugs or exhibit other fascinating biological activities.^4^ Their biosynthesis is often executed by type II nonreducing polyketide synthases (NR‐PKSs) in bacteria and iterative type I NR‐PKS in fungi that catalyze the Claisen‐type condensation of acetyl‐CoA and malonyl thioesters to yield a linear polyketide that undergoes regioselective cyclization and/or aromatization. The products are further processed by tailoring enzymes to implant post‐aromatic modifications, thus creating molecular diversity.^1^, ^5^, ^6^ Despite the occurrence of similar metabolites, such as tetrahydroxynaphthalene (T_4_HN), both in bacteria^7^ and fungi,^8^ they have been shown to arise by different downstream processing routes during biosynthesis.^9^

In another example, naphthohydroquinones are formed either through two‐electron reduction of naphthoquinones,^10^ or through tautomerization of a monoreduced hydroxynaphthoquinone by fungal tetrahydroxynaphthalene reductase (T_4_HNR).^11^ A major difference apparently occurs at the reduction step. In bacteria, the reduction of a carbonyl group by an NADPH‐dependent ketoreductase (KR) is believed to be carried out on a linear polyketide chain before cyclization and aromatization^1^, ^2^ or on a monocyclized derivative after the first cyclization and dehydration.^12^ In contrast, fungal enzymes reduce fully aromatized substrates, for example, during melanin biosynthesis^9^, ^13^ or monodictyphenone biosynthesis.^14^

GTRI‐02 (**2**), a partly reduced, bicyclic polyketide, is produced by diverse bacteria. It was first isolated from the soil actinomycete *Micromonospora* sp. SA246 and exhibits antioxidant properties.^15^ GTRI‐02 is also produced by *Streptomyces* sp. strains GW4184,^16^ ANK313,^17^ and Gö C4/4,^18^, ^19^ and recently was identified in *Streptomyces violaceoruber* 20 and *Streptomyces coelicolor* A3(2).^21^ According to a bio‐retrosynthetic analysis (using fungal biogenesis), **2** is synthesized chemoenzymatically by use of a fungal enzyme. This was achieved by the regio‐ and stereoselective reduction of acetyltrihydroxynaphthalene (AcT_3_HN, **1**) with the NADPH‐dependent T_4_HNR from *Magnaporthe grisea* (Scheme 1, path A).^22^ The corresponding reduction step in bacterial biosynthesis is still unknown; in particular, it is not known whether a linear octaketide **3**, a cyclic nonaromatic precursor, or trihydroxynaphthalene **1** is the actual substrate. One might assume that the biosynthesis of **2** in bacteria also involves the reduction of aromatic substrate **1**, a strategy we have successfully applied in its total synthesis.^22^ However, more recently, **2** has been identified as an additional product of the *act* gene cluster in *S. coelicolor* A3(2).^21^

**Scheme 1:**
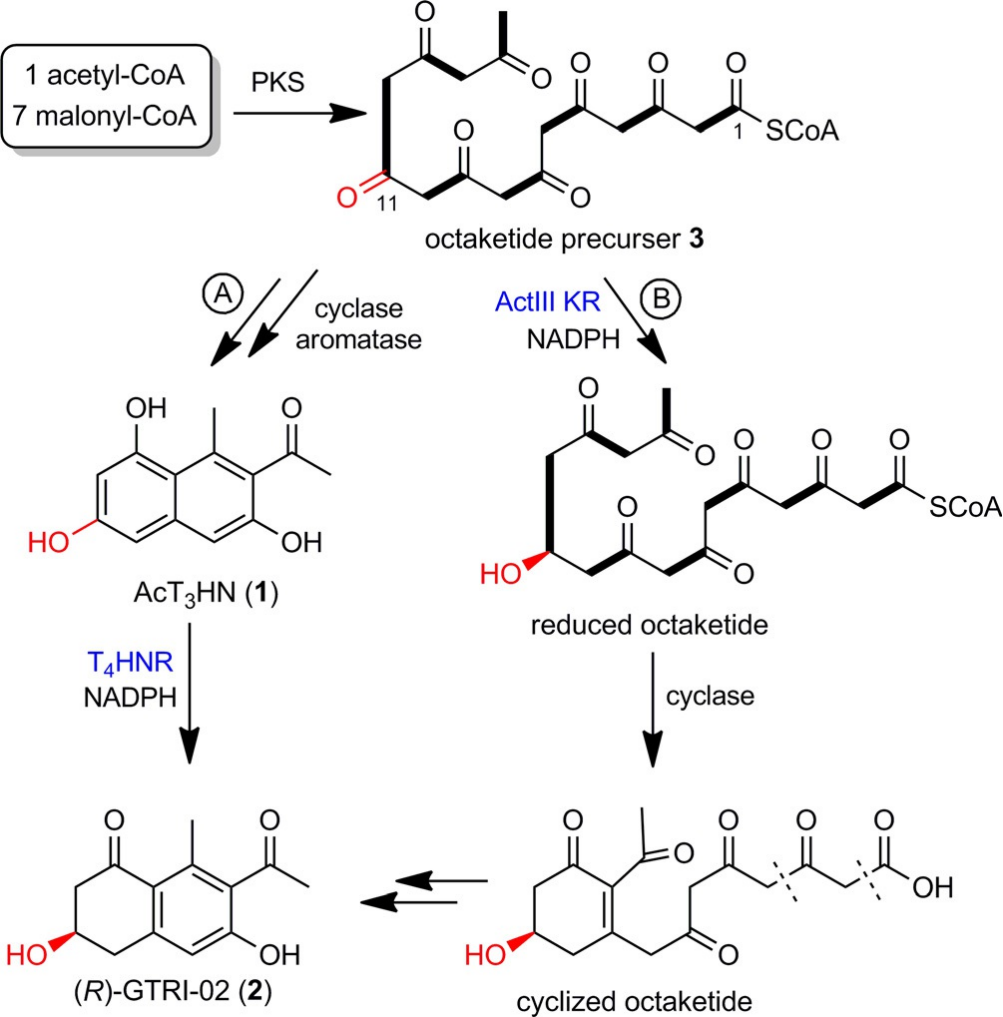
Proposed routes for the biosynthesis of GTRI‐02 (**2**) according to A) ref. 22 and B) ref. 21.

The corresponding ketoreductase is ActIII KR, which is supposed to reduce linear octaketide **3** prior to cyclization and aromatization (Scheme 1, path B).^21^ Herein, we resolve the issue pertaining to the substrate of bacterial KRs during the biosynthesis of GTRI‐02 by testing three bacterial KRs with mono‐, bi‐, and tricyclic aromatic substrates.

The (*R*)‐**2** producer strain *Streptomyces* sp. GW4184 was obtained from Prof. Hartmut Laatsch (University of Göttingen). To verify metabolite production, the strain was grown according to the literature.^16^ After 3 days, ethyl acetate extracts were analyzed for **2** and the aromatized precursor **1** by LC‐MS by comparison with authentic samples obtained by synthesis.^22^ Although the production of **2** was confirmed, compound **1** could not be detected.

To identify the gene cluster and the enzymes responsible for the production of **2**, we sequenced the genome of *Streptomyces* sp. GW4184. Sequence analysis did not show the presence of a putative T_4_HNR‐like enzyme in the genome. Nevertheless, genome analysis revealed two PKS type II gene clusters containing two different putative ketoreductases “KR1” (contig 220‐ORF9) and “KR2” (contig 313‐ORF14), which might be responsible for the reduction step in the biosynthesis of **2**. Comparison of T_4_HNR (*M. grisea*)^24^ with KR1, KR2, and the known bacterial ketoreductases ActIII KR from *S. coelicolor* A3(2),^25^ msn KR from *Streptomyces* sp. Gö C4/4,^18^ KR from *Streptomyces fradiae*,^27^ and julichrome KR (Juli) from *Streptomyces afghaniensis* NC5228^28^ gave only 27–30 % sequence identity. However, the bacterial enzymes share 59–71 % sequence identity with each other (Tables S1 and S2 in the Supporting Information). All enzymes show the presence of an NAD(P)H binding pocket recognized as a Rossmann fold (Figure 1).^29^ The sequence alignment further shows that active‐site residues (Asn130, Ser156, Tyr170, Lys174 in T_4_HNR;^24^ Asn114, Ser144, Tyr157, Lys161 in ActIII KR^25^) remain conserved in all the enzymes; this supports their function as short‐chain dehydrogenases/reductases (SDRs). However, the amino acid residues involved in binding of a putative substrate did not match between the fungal and bacterial enzymes, thus indicating that the two enzyme types might catalyze the reduction of different physiological substrates. These findings prompted us to test various aromatic substrates **1** and **4**–**15** for reduction by bacterial KRs (Scheme 2). Linear polyketide chains of corresponding lengths cannot be tested due to their intrinsically low stability.^30^

**Figure 1:**
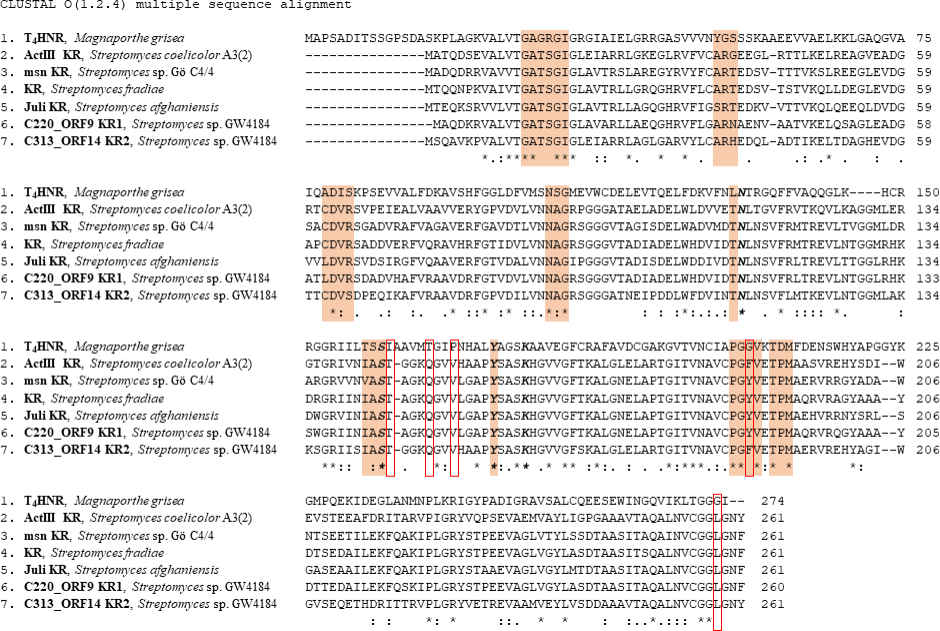
Multiple sequence alignment (CLUSTAL Omega 1.2.4)^23^ of amino acid sequences of T_4_HNR and various ketoreductases. Three‐dimensional information for T_4_HNR (PDB ID: 1JA9)^24^ and ActIII KR (PDB ID: 1W4Z)^25^ was accessed by using iCn3D Structure Viewer^26^ at NCBI. All mentioned proteins share an NAD(P)H binding site (shaded) and identical catalytic residues (bold and italics) at the active site. However, T_4_HNR differs from the KRs by key substrate binding residues (boxed), which might impart functional differences.

**Scheme 2:**
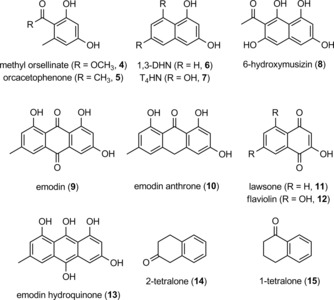
Substrates **4**–**15** tested for reduction by KR1, KR2 from *Streptomyces* sp. GW4184, and ActIII KR from *S. coelicolor* A3(2).

For this purpose, KR1 and KR2 as well as one of the best‐studied bacterial enzymes of polyketide reduction, ActIII KR, were chosen.^25^ The genes were cloned into a pET19b vector and expressed in *Escherichia coli* BL21(DE3) cells. The N‐terminally His‐tagged proteins were purified by using Ni‐NTA affinity chromatography (Supporting Information). First, reduction of the proposed biosynthetic substrate, **1**, was tested with the three enzymes. None of the bacterial KRs could reduce **1** using NADPH, whereas T_4_HNR is known to catalyze this transformation (Scheme 1).^22^ This suggests that an alternative biosynthetic route to **2** operates in bacteria. To further explore the catalytic promiscuity of the three bacterial KRs, mono‐, bi‐, and tricylic compounds **4**–**15** were tested as substrates (Scheme 2).

Compounds **4**–**15** were chosen based on their polyketide origin and the ability of fungal and bacterial enzymes belonging to the SDR family to reduce some of these compounds by using NADPH. They were obtained from commercial sources or synthesized (Supporting Information). Of these substrates, only the bicyclic compounds 2‐tetralone (**14**) and 1‐tetralone (**15**) were reduced by KR1, KR2, and ActIII KR. Not accepted as substrates were methyl orsellinate (**4**) and orcacetophenone (**5**), representing monocyclic aromatic tetraketides, polyhydroxynaphthalenes **6**–**8**,^13^ tricyclic aromatic emodin (**9**) and emodin anthrone (**10**), the hydroxynaphthoquinones, lawsone (**11**) and flaviolin (**12**), and emodin hydroquinone (**13**; formed in situ from emodin by Na_2_S_2_O_4_). Several of these compounds (**6**–**8**, **11**–**13**) are known to be reduced by T_4_HNR^22^ or related enzymes such as MdpC from *Aspergillus nidulans*.^9^ To regenerate NADPH, l‐malic acid and malate dehydrogenase (decarboxylating, MAE) were used, as glucose dehydrogenase is known to catalyze the reduction of tetralones.^13^ KR1 from *Streptomyces* sp. GW4184 showed quantitative conversion of **14** into 2‐tetralol (**16**), yet only 20 % conversion of **15** into 1‐tetralol (**17**), as analyzed by ^1^H NMR spectroscopy (Table 1, entry 1). Likewise, KR2 and ActIII KR from *S. coelicolor* A3(2) reduced **14** to **16** with 13 % and 30 % conversion when using NADPH. Application of both enzymes resulted in little conversion of 1‐tetralone (**15**; Table 1, entries 2 and 3).

**Table 1:** Enzymatic products obtained by the reduction of tetralones **14** and **15**.

	Enzyme^[a]^	% Conversion (^1^H NMR)	
		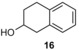	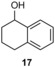
1	KR 1	>99	20
2	KR2	13	<2
3	ActIII KR	30	<5

[a] NADPH was regenerated by using l‐malic acid and MAE.

The ability of KR1 to reduce **14** and **15** is in accordance with earlier studies on ActIII KRs demonstrating catalytic reduction of bicyclic compounds such as tetralones or decalones rather than linear and monocyclic substrates.^31^ In a screening with a number of substrates including decalones, tetralones, and substituted cyclohexane‐1,3‐diones, Korman et al. found that ActIII KR had no activity with monocyclic ketones, acetoacetyl‐CoA, or acetoacetyl‐ACP. Instead, the bicyclic ketone substrates *trans*‐1‐decalone, 2‐decalones, and 1‐tetralone (**15**) were reduced.^31^, ^32^ These results correspond with our observations and suggest that in the biosynthesis of **2** (and its deoxy compound **22**) cyclic, but not yet aromatic, intermediates, such as **18** and **19**, undergo a KR‐catalyzed reduction. For the biosynthesis of deoxygenated aromatic bacterial polyketides, we propose that a linear polyketide chain undergoes a first cyclization through aldol reaction/condensation before it is reduced by a KR and prior to further cyclizations, aromatization, or other tailoring steps (Scheme 3). Formation of the deoxy compound **22** is probably due to dehydratase activity on either the reduced monocyclic compound **20** or the final **2**, as is the case in the biosynthesis of 1,8‐dihydroxynaphthalene.^9^

**Scheme 3:**
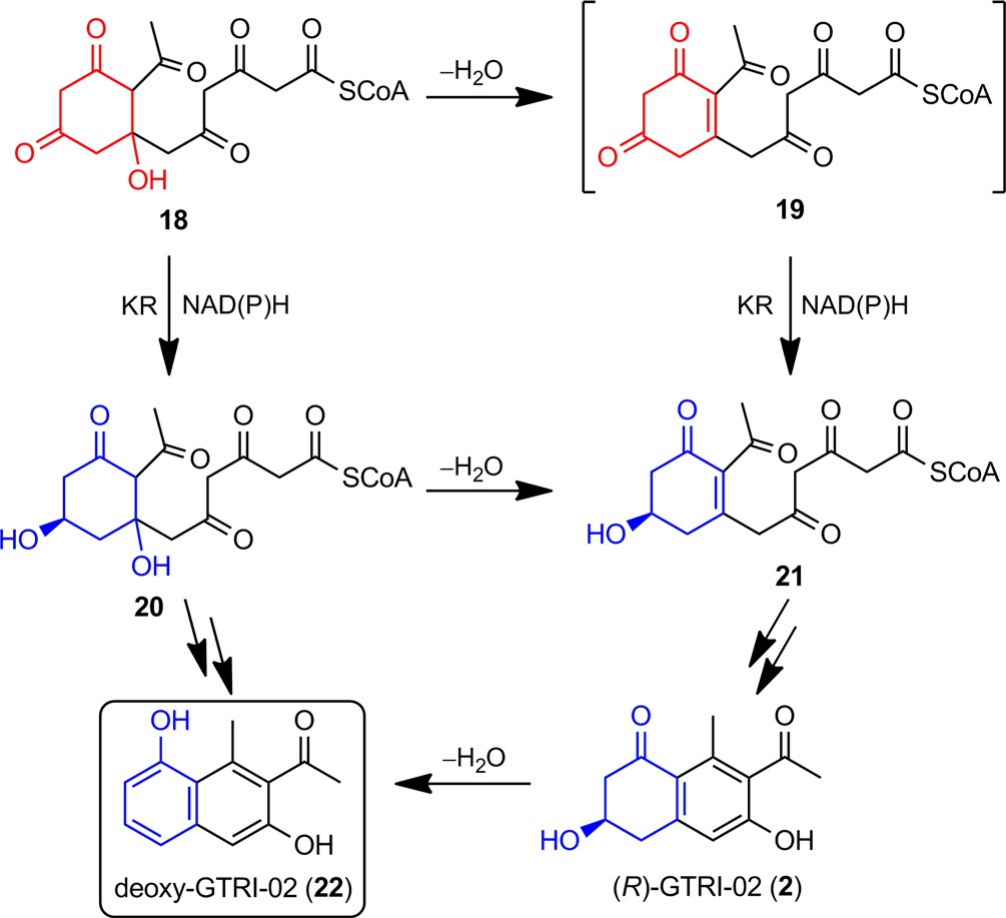
Proposed biosynthesis of the aromatic polyketide GTRI‐02 (**2**) in *Streptomyces* spp.

Hence, the substrate for the KR in the biosynthesis of bacterial (*R*)‐**2** is probably neither a fully aromatic naphthol (**1**) nor a linear polyketide (**3**) as proposed previously.^21^, ^22^ Nevertheless, Funa and co‐workers identified an aldo‐keto reductase (AKR), but not an SDR, in the myxobacterium *Sorangium cellulosum* catalyzing the reduction of T_4_HN and T_3_HN (Scheme 4 B).^33^ Furthermore, they demonstrated the lack of any enzyme from *S. coelicolor* able to catalyze such reductions; again this is in accordance with our observations.

**Scheme 4:**
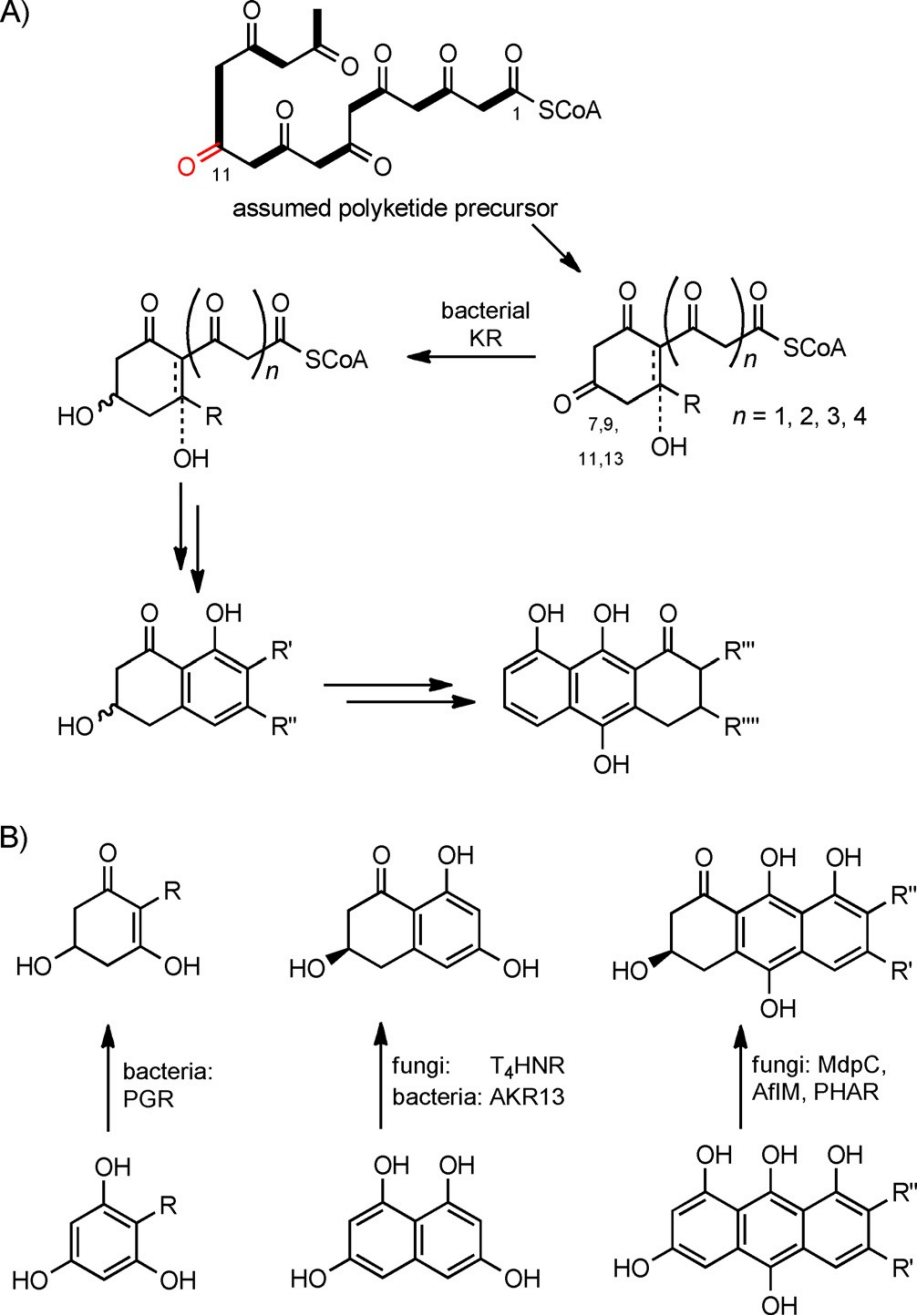
A) Proposed enzymatic reduction of polyketide‐derived monocyclic 1,3‐diones by a bacterial KR, followed by, for example, cyclization, aromatization, dehydration, and oxidation; B) Enzymatic reduction of mono‐, bi‐, and tricyclic aromatic polyketides by bacterial and fungal SDRs and a bacterial AKR. PGR: phloroglucinol reductase; PHAR: polyhydroxyanthracene reductase.

Moreover, the previously assumed “position‐controlled” carbonyl reduction of a longer polyketide chain (such as at C11 of **3**, Scheme 1, path B) can be excluded due to the low stability of long‐chain polyketides.^30^ Instead of a “position‐controlled” reduction of a longer polyketide, our results are congruent with an enzymatic reduction of a first‐cyclized intermediate, as shown in a generalized form in Scheme 4 A. Subsequent cyclization, aromatization, dehydration, and oxidation will give the mono‐, bi‐, tri‐, and polycyclic (deoxygenated) aromatic natural products.

This approach is complemented by alternative enzyme‐catalyzed reductions of mono‐, bi‐, and tricyclic polyhydroxylated aromatic compounds (Scheme 4 B).^9^, ^34^ Hence, although the final (deoxygenated) polyketide natural products from bacteria and fungi can be similar or even identical (compare products Scheme 4 A and B), their biosynthetic pathways can be quite different.

## Conflict of interest

*The authors declare no conflict of interest*.

## Supporting information

As a service to our authors and readers, this journal provides supporting information supplied by the authors. Such materials are peer reviewed and may be re‐organized for online delivery, but are not copy‐edited or typeset. Technical support issues arising from supporting information (other than missing files) should be addressed to the authors.

Supplementary
