# Supplementary material for: Insights into the Role of Ketoreductases in the Biosynthesis of Partially Reduced Bacterial Aromatic Polyketides*
Source: Chembiochem. 2019 Dec 9;21(6):780–4. doi: 10.1002/cbic.201900357 (PMC7154522; doi:10.1002/cbic.201900357)
Supplement: Supplementary file 1 — Supplementary [file CBIC-21-780-s001.pdf]

CHEMBIOCHEM

Supporting Information

# Insights into the Role of Ketoreductases in the Biosynthesis of Partially Reduced Bacterial Aromatic Polyketides*

Syed Masood Husain+,[a] Andreas Präg+,[b] Anton Linnenbrink,[c] Andreas Bechthold,[c] and Michael Müller*[b]

cbic_201900357_sm_miscellaneous_information.pdf

# Supporting Information


I. General remarks S2

II. Sequence alignment of $T_4$HNR with bacterial enzymes S4–S4

III. Molecular cloning, bacterial expression, and activity measurements S4–S8

IV. Synthesis of substrates S9

V. Enzymatic reduction of substrates **1** and **4**–**15** S9–S12

VI. References S13

## I. General remarks

All chemical reagents and solvents were obtained from Sigma Aldrich and HiMedia. NMR spectra were recorded at 24 °C on a DRX 400 spectrometer (Bruker) operating at 400 and 100 MHz for $^{1}H$ and $^{13}C$ acquisitions, respectively. Chemical shifts ($\delta$) of the $^{1}H$ and $^{13}C$ NMR spectra are reported in ppm with a solvent resonance as internal standard ($^{1}H$ NMR: $CHCl_3$ 7.26, acetone-$d_6$ 2.05; $^{13}C$ NMR: $CDCl_3$ 77.16, acetone-$d_6$ 29.84). Coupling constants ($J$) are given in Hz. GC-MS analysis was carried out on an HP 6890N Series GC system (EI, 70 eV) equipped with an HP 5973 Network Mass Selective Detector (Agilent), using an FS-Supreme-5 column (L = 30 m, diameter = 0.25 mm, film = 0.25 µm; CS-Chromatographie Service). The temperature gradient $T_{0–3\ min}$ = 60 °C, $T_{14\ min}$ = 280 °C, $T_{19\ min}$ = 280 °C was used. Enzymatic assays and UV Spectra were recorded on a UV Mini 1240 UV/Vis spectrophotometer from Shimadzu. PCR amplification was performed using a Perkin Elmer Gene Amp PCR system 2400 and a gradient PCR cycler (Eppendorf). Glucose dehydrogenase (GDH) and malic enzyme (MAE, malate dehydrogenase, decarboxylating) were obtained from evocatal. Yields refer to chromatographically pure materials; conversions were calculated from reactant–product ratios in crude $^{1}H$ NMR spectra.

## II. Sequence alignment of $T_4$HNR with bacterial enzymes

### Protein sequence for KR1_C220_ORF9 (*Streptomyces* sp. GW4184)

MAQDKRVALVTGATSGIGLAVARLLAEQGHRVFLGARNAENVAATVKELQSAGLEADGATLDVRSD
ADVHAFVRAAVDRFGTVDVLVNNAGRSGGGVTADIADELWHDVIDTNLNSVFRLTREVLNTGGLRH
KSWGRIINIASTAGKQGVVLGAPYSASKHGVVGFTKALGNELAPTGITVNAVCPGYVETPMAQRVRQG
YAAAYDTTEDAILEKFQSKIPLGRYSTPEEVAGLVGYLASDTAASITAQALNVCGGLGNF

### Protein sequence for KR2_C313_ORF14 (*Streptomyces* sp. GW4184)

MSQAVKPVALVTGATSGIGLEIARRLAGLGARVYLCARHEDQLADTIKELTDAGHEVDGTTCDVSDPE
QIKAFVRAAVDRFGPVDILVNNAGRSGGGATNEIPDDLWFDVINTNLNSVFLMTKEVLNTGGMLAKKS
GRIISIASTGGKQGVVHAAPYSASKHGVVGFSKALGLELARTGITVNAVCPGFVETPMAERVREHYAGI
WGVSEQETHDRITTRVPLGRYVETREVAAMVEYLVSDDAAAVTAQALNVCGGLGNY

### Protein sequence for ActIII_KR (*Streptomyces coelicolor* A3(2))

MATQDSEVALVTGATSGIGLEIARRLGKEGLRVFVCARGEEGLRTTLKELREAGVEADGRTCDVRSVP
EIEALVAAVVERYGPVDVLVNNAGRPGGGATAELADELWLDVVETNLTGVFRVTKQVLKAGGMLER
GTGRIVNIASTGGKQGVVHAAPYSASKHGVVGFTKALGLELARTGITVNAVCPGFVETPMAASVREHY
SDIWEVSTEEAFDRITARVPIGRYVQPSEVAEMVAYLIGPGAAAVTAQALNVCGGLGNY

### Protein sequence for Juli_KR (*Streptomyces afghaniensis* NC5228)

MTEQKSRVVLVTGATSGIGLAVTRLLAGQGHRVFIGSRTEDKVVTTVKQLQEEQLDVDGVVLDVRSV
DSIRGFVQAAVERFGTVDALVNNAGIPGGGVTADISDELWDDIVDTNLNSVFRLTREVLTTGGLRHKD
WGRVINIASTAGKQGVVLGAPYSASKHGVVGFTKALGNELAPTGITVNAVCPGYVETPMAEHVRRNY
SRLSGASEAAILEKFQAKIPLGRYSTAAEVAGLVGYLMTDTAASITAQALNVCGGLGNF

### Protein sequence for KR (*Streptomyces fradiae*)

MTQQNPKVAIVTGATSGIGLAVTRLLGRQGHRVFLCARTEDSVTSTVKQLLDEGLEVDGAPCDVRSAD
DVERFVQRAVHRFGTIDVLVNNAGRSGGGVTADIADELWHDVIDTNLNSVFRMTREVLNTGGMRHK
DRGRIINIASTAGKQGVVLGAPYSASKHGVVGFTKALGNELAPTGITVNAVCPGYVETPMAQRVRAGY
AAAYDTSEDAILEKFQAKIPLGRYSTPEEVAGLVGYLASDTAASITSQALNVCGGLGNF

### Protein sequence for msn_KR (*Streptomyces bottropensis* st. Gö C4/4)

MADQDRRVAVVTGATSGIGLAVTRSLAREGYRVYFCARTEDSVTTTVKSLREEGLEVDGSACDVRSG
ADVRAFVAGAVERFGAVDTLVNNAGRSGGGVTAGISDELWADVMDTNLNSVFRMTREVLTVGGML
DRARGRVVNVASTAGKQGVVLGAPYSASKHGVVGFTKALGNELAPTGITVNAVCPGYVETPMAERV
RRGYADAWNTSEETILEKFQAKIPLGRYSTPEEVAGLVTYLSSDTAASITAQAINVCGGLGNF

**Table S1. Sequence alignment of bacterial ketoreductases with fungal tetrahydroxy-naphthalene reductase ($T_4$HNR, UniProt ID: Q9HFV6) of *Magnaporthe grisea*.**

| Entry | Enzyme (Source Organism) | % Identity (BLASTp) | E value |
|---|---|---|---|
| 1 | KR1_C220_ORF9 (*Streptomyces* sp. GW4184) | 29.77 | 1e-26 |
| 2 | KR2_C313_ORF14 (*Streptomyces* sp. GW4184) | 29.55 | 4e-30 |
| 3 | ActIII_KR (*Streptomyces coelicolor* A3(2)) | 30.20 | 2e-26 |
| 4 | Juli_KR (*Streptomyces afghaniensis* NC5228) | 27.10 | 4e-23 |
| 5 | KR (*Streptomyces fradiae*) | 28.63 | 8e-24 |
| 6 | msn_KR (*Streptomyces bottropensis* st. Gö C4/4) | 27.91 | 1e-23 |

**Table S2. Sequence alignment of bacterial ketoreductases with ActIII_KR from *Streptomyces coelicolor* A3(2).**

| Entry | Enzyme (Source Organism) | % Identity (BLASTp) | E value |
|---|---|---|---|
| 1 | KR1_C220_ORF9 (*Streptomyces* sp. GW4184) | 62.02 | 3e-118 |
| 2 | KR2_C313_ORF14 (*Streptomyces* sp. GW4184) | 70.50 | 7e-136 |
| 3 | Juli_KR (*Streptomyces afghaniensis* NC5228) | 59.39 | 1e-111 |
| 4 | KR (*Streptomyces fradiae*) | 60.15 | 9e-119 |
| 5 | msn_KR (*Streptomyces bottropensis* st. Gö C4/4) | 63.60 | 1e-124 |

## III. Molecular cloning, bacterial expression, and activity measurements

*Bacteria, DNA preparation, and expression vector*

*E. coli* SIG10 and BL21(DE3) (Sigma Aldrich) were used for cloning and expression of KR1, KR2, and ActIII KR. The expression vector pET-19b was obtained from Novagen. *Streptomyces* sp. GW4184 culture was provided by Prof. H. Laatsch, University of Göttingen, Germany, and sequenced by BASECLEAR. The two ketoreductases KR1 (Contig 220-ORF9) and KR2 (Contig 313-ORF14) of the two type II PKS from *Streptomyces* sp. GW4184 were cloned into the pET-19b vector from its genomic DNA using the In-Fusion Cloning Kit from Clontech Laboratories. Similarly, ActIII KR of the actinorhodin biosynthesis gene cluster from *Streptomyces coelicolor* A3(2) was cloned using genomic DNA.

*Cloning and sequence analysis*

For the cloning of the ketoreductase genes into pET19b vector the In-Fusion HD Cloning Kit from Clontech Laboratories was used. First, the vector was linearized by digestion with *Xho*I. Then, the respective genes were cloned from genomic DNA using primers, which had a 5'-end complementary for the respective ends of the linearized vector, and a 3'-end complementary to the start or end of the gene of interest. Furthermore, the originally included *Xho*I cut site was preserved. After In-Fusion cloning, the resulting circular plasmids were transformed by heat shock at 42 °C for 45 s into DH5α cells, which were consequently grown under selective pressure (ampicillin). Correct sequences and orientation of the amplified genes in the vector were confirmed by sequence analysis through GATC Biotech.

***Primers for cloning of KR1 of* Streptomyces *sp. GW4184***

C220_ORF9_for CAAGCATATGCTCGAGATGGCGCAGGACAAGC

C220_ORF9_rev CAGCCGGATCCTCGAGTCAGAAGTTGCCGAGGC

***Primers for cloning of KR2 of* Streptomyces *sp. GW4184***

C313_ORF14_for CAAGCATATGCTCGAGATGTCACAGGCAGTCAAGCC

C313_ORF14_rev CAGCCGGATCCTCGAGTCAGTAGTTGCCCAGACCG

***Primers for cloning ActIII_KR of* Streptomyces coelicolor *A3(2)***

ActIII_KR_for CAAGCATATGCTCGAGATGGCCACGCAGGACTC

ActIII_KR_rev CAGCCGGATCCTCGAGATGTCACAGGCAGTCAAGCC

*Media and growth conditions*

One clone was picked and suspended in 5 mL of LB medium (Lennox) containing ampicillin (100 µg·mL$^{-1}$), followed by incubation for 8 h (37 °C, 160 rpm). The culture was dosed with glycerol (20% v/v) and stored at –20 °C. For use, 20 µL of this culture was then suspended in 10 mL of LB medium (Lennox) containing ampicillin (100 µg·mL$^{-1}$), followed by incubation overnight (37 °C, 160 rpm).

*Cultivation and expression*

The overnight cultures were diluted to 500 mL of medium each (ampicillin 100 µg·mL$^{-1}$). IPTG (0.1 mM) was added after the mid-log phase ($OD_{600nm}$ = 0.6) was reached. The cultures were incubated for 20 h at 25 °C and 160 rpm.

*Workup and storage*

The harvested *E. coli* cells were resuspended in lysis buffer (50 mM Tris HCl, 5 mM imidazole, 0.5 mM DTT, 10% glycerol, pH 7.5; 2.5 mL per harvested cells of 500 mL medium). The cells were disrupted by sonication (6 × 15 s; Branson Sonifier II, Model W-250, Heinemann), followed by centrifugation (20 min, 12000 × *g*, 4 °C). Glycerol (20% v/v) was added and the crude enzyme preparation was frozen at –20 °C.

*Enzyme purification*

KR1-his, KR2-his, and ActIII_KR-his were purified by Ni-NTA affinity chromatography. Non-specifically bound proteins were washed off with washing buffer (50 mM Tris HCl, 20 mM imidazole, 10% glycerol, pH 7.5). Elution was performed with an elution buffer (50 mM Tris HCl, 250 mM imidazole, 10% glycerol, pH 7.5). The eluted solution was desalted by gel filtration (Sephadex™ G-25M, GE Healthcare). Concentration of the protein was performed by ultrafiltration (Amicon Ultra-15 Centrifugal Filter Unit, 10000 NMWL, Merck Millipore).

## Nucleotide and protein sequences

*Nucleotide sequence of KR1 (*Streptomyces *sp. GW4184_C220_ORF9)* **783 bp**

ATGGCGCAGGACAAGCGGGTCGCCCTGGTGACCGGGGCGACCAGCGGCATCGGTCTGGCCGTGGC
CCGGCTGCTGGCCGAACAGGGGCACCGGGTGTTCCTGGGCGCGCGCAACGCCGAGAACGTGGCCG
CGACCGTGAAGGAGCTCCAGAGCGCCGGCCTGGAGGCGGACGGCGCCACGCTGGACGTCCGCTCC
GACGCCGACGTGCACGCGTTCGTGCGGGCGGCCGTGGACCGGTTCGGCACGGTGGACGTGCTGGT
GAACAACGCCGGCCGCAGTGGCGGCGGGGTGACCGCGGACATCGCCGACGAGCTGTGGCACGAC
GTGATCGACACCAACCTCAACAGCGTGTTCCGGCTGACCCGCGAGGTGCTCAACACCGGCGGCCT
GAGGCACAAGAGCTGGGGCCGGATCATCAACATCGCGTCCACCGCGGGCAAGCAGGGCGTCGTCC
TGGGCGCCCCGTACTCGGCGTCCAAGCACGGCGTGGTCGGCTTCACCAAGGCCCTCGGCAACGAG
CTGGCCCCGACCGGCATCACCGTCAACGCGGTCTGCCCGGGCTACGTCGAGACGCCGATGGCCCA
GCGGGTGCGCCAGGGCTACGCCGCCGCCTACGACACCACCGAGGACGCGATCCTGGAGAAGTTCC
AGTCCAAGATCCCGCTCGGCCGCTACTCCACCCCCGAGGAGGTCGCAGGGCTGGTCGGCTACCTG
GCCTCCGACACCGCCGCCTCCATCACCGCGCAGGCGCTCAACGTCTGCGGCGGCCTCGGCAACTTC
TGA

*Nucleotide sequence of KR2 (*Streptomyces *sp. GW4184_C313_ORF14)* **786 bp**

ATGTCACAGGCAGTCAAGCCGGTCGCCCTGGTCACGGGAGCCACCAGCGGGATCGGTCTCGAGAT
CGCCCGGCGCCTCGCCGGGCTCGGGGCCCGGGTCTACCTGTGTGCGCGCCATGAGGACCAACTGG
CCGACACCATCAAGGAGTTGACGGACGCGGGGCACGAGGTGGACGGCACCACGTGCGATGTGTCC
GACCCCGAGCAGATCAAGGCCTTCGTCCGCGCGGCCGTCGACCGCTTCGGGCCGGTCGACATCCTC
GTCAACAACGCCGGGCGCAGCGGCGGCGGAGCCACCAATGAGATCCCGGACGACCTCTGGTTCGA
TGTCATCAACACGAACCTCAACAGTGTCTTCCTGATGACCAAGGAAGTCCTCAACACCGGTGGCAT
GCTGGCGAAGAAGTCCGGCCGGATCATCTCGATCGCCTCCACCGGCGGCAAGCAGGGCGTCGTGC
ACGCCGCGCCCTACTCGGCGTCCAAGCACGGTGTGGTCGGCTTCTCCAAGGCTCTCGGTCTGGAGC
TGGCCCGTACCGGCATCACGGTCAACGCGGTGTGCCCCGGCTTCGTCGAGACGCCGATGGCGGAG
CGGGTGCGCGAGCACTACGCCGGCATCTGGGGCGTGAGCGAGCAGGAGACCCACGACCGCATCAC
CACGCGGGTGCCGCTGGGCCGTTACGTGGAGACCCGTGAGGTGGCCGCCATGGTCGAGTACCTGG
TGAGCGACGACGCGGCCGCGGTGACCGCGCAGGCCCTCAACGTGTGCGGCGGTCTGGGCAACTAC
TGA

*Nucleotide sequence of ActIII KR (*Streptomyces coelicolor *A3(2)_ActIII)* **786 bp**

ATGGCCACGCAGGACTCCGAAGTCGCACTGGTGACGGGTGCGACCAGCGGAATCGGGCTGGAGAT
CGCCCGCAGGCTCGGTAAGGAGGGGTTGCGCGTGTTCGTCTGCGCGCGCGGCGAAGAAGGACTGC
GGACGACGCTGAAGGAGTTGCGGGAGGCGGGCGTGGAGGCGGACGGGCGGACCTGCGACGTCCG
CTCGGTCCCGGAGATCGAGGCTCTCGTGGCGGCGGTGGTCGAGCGTTACGGTCCGGTCGACGTGCT
GGTGAACAACGCGGGCCGGCCCGGCGGCGGCGCCACGGCCGAACTCGCCGACGAACTCTGGCTCG
ATGTCGTGGAGACCAACCTCACCGGCGTGTTCCGGGTGACCAAACAGGTCCTCAAGGCGGGCGGC
ATGCTCGAACGGGGCACGGGCCGAATCGTCAACATCGCCTCGACCGGCGGAAAGCAGGGTGTGGT
GCACGCCGCGCCCTACTCCGCCTCGAAGCACGGCGTGGTCGGCTTCACCAAGGCGCTCGGTCTCGA

ACTGGCGAGGACCGGCATCACGGTGAACGCCGTCTGCCCCGGATTCGTCGAGACGCCGATGGCCG
CGTCCGTGCGCGAGCACTACTCGGACATCTGGGAGGTGTCGACCGAGGAGGCCTTCGACCGGATC
ACCGCGCGCGTGCCGATCGGCCGGTACGTGCAGCCGTCCGAGGTGGCGGAGATGGTGGCGTACCT
GATCGGGCCCGGTGCGGCCGCGGTCACCGCGCAGGCGCTGAACGTCTGCGGCGGGCTGGGGAACT
ACTGA

*Protein sequence of KR1-his*

MGHHHHHHHHHHSSGHIDDDDKHMAQDKRVALVTGATSGIGLAVARLLAEQGHRVFLGARNAENV
AATVKELQSAGLEADGATLDVRSDADVHAFVRAAVDRFGTVDVLVNNAGRSGGGVTADIADELWHD
VIDTNLNSVFRLTREVLNTGGLRHKSWGRIINIASTAGKQGVVLGAPYSASKHGVVGFTKALGNELAPT
GITVNAVCPGYVETPMAQRVRQGYAAAYDTTEDAILEKFQSKIPLGRYSTPEEVAGLVGYLASDTAASI
TAQALNVCGGLGNF

*Protein sequence of KR2-his*

MGHHHHHHHHHHSSGHIDDDDKHMSQAVKPVALVTGATSGIGLEIARRLAGLGARVYLCARHEDQL
ADTIKELTDAGHEVDGTTCDVSDPEQIKAFVRAAVDRFGPVDILVNNAGRSGGGATNEIPDDLWFDVI
NTNLNSVFLMTKEVLNTGGMLAKKSGRIISIASTGGKQGVVHAAPYSASKHGVVGFSKALGLELARTG
ITVNAVCPGFVETPMAERVREHYAGIWGVSEQETHDRITTRVPLGRYVETREVAAMVEYLVSDDAAA
VTAQALNVCGGLGNY

*Protein sequence of ActIII_KR-his*

MGHHHHHHHHHHSSGHIDDDDKHMATQDSEVALVTGATSGIGLEIARRLGKEGLRVFVCARGEEGLR
TTLKELREAGVEADGRTCDVRSVPEIEALVAAVVERYGPVDVLVNNAGRPGGGATAELADELWLDVV
ETNLTGVFRVTKQVLKAGGMLERGTGRIVNIASTGGKQGVVHAAPYSASKHGVVGFTKALGLELART
GITVNAVCPGFVETPMAASVREHYSDIWEVSTEEAFDRITARVPIGRYVQPSEVAEMVAYLIGPGAAAV
TAQALNVCGGLGNY

## IV. Synthesis of substrates

Substrates used in the work were either purchased commercially or synthesized as per the procedure reported elsewhere.[1–6]

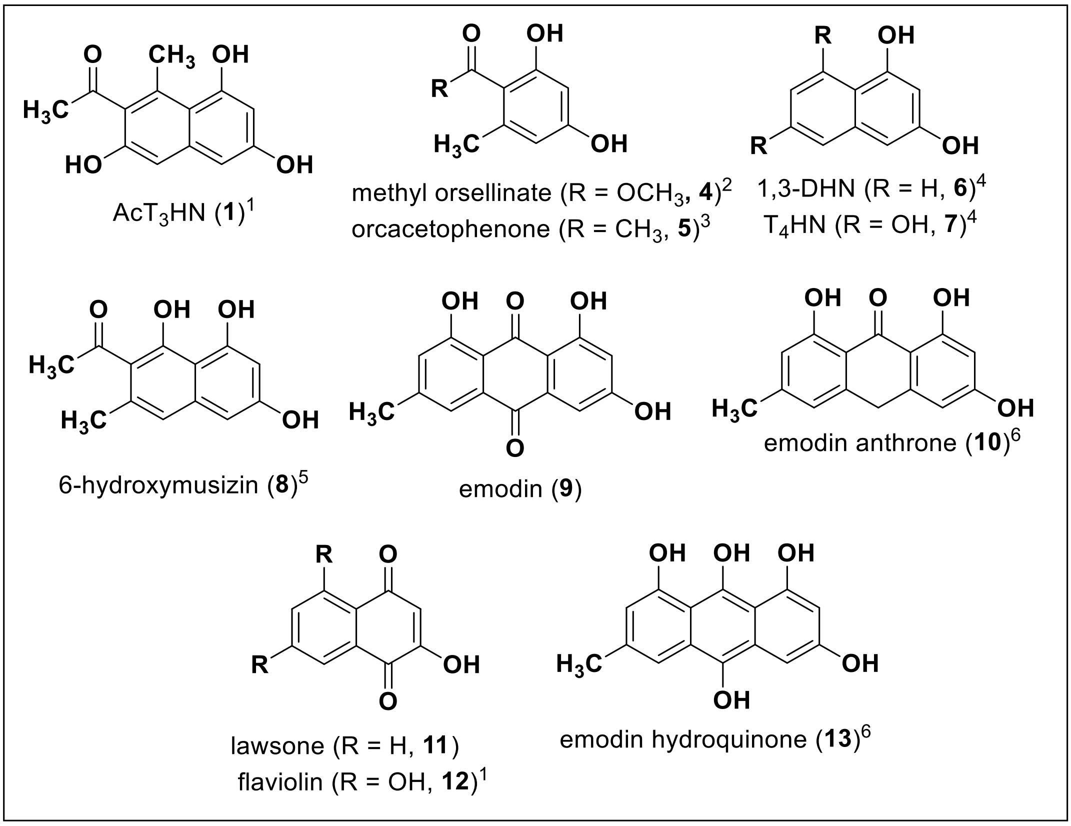


**Figure S1.** Substrates tested with KR1_his, KR2_his and ActIII KR_his.

## V. Enzymatic reduction of substrates 1 and 4–15

**General Procedure for enzymatic reduction:** To a buffer solution (10.0 mL; 50 mM KPi, 1 mM EDTA, 1 mM DTT, pH 7.0), a current of argon was bubbled for 30 min. Then, glucose (5.0 equiv), $NADP^+$ (0.1 equiv, 10 mol%), and glucose dehydrogenase (400 µL, 150 U/mL) were added, and the mixture was stirred slowly under argon at rt. After 1 h, the substrate, (**1, 4–12**; 136 µmol, 1.0 equiv), in 2-propanol (0.5 mL, 5% v/v) was added slowly, while stirring. For formation of **13** *in situ* $Na_2S_2O_4$ (20 equiv) was added to emodin (1 equiv.). Lastly, the enzyme purified enzyme (KR1_his, KR2_his or ActIII KR_his (2 mL) was added, and the mixture was stirred at rt. After 24 h, the solution was acidified with 10% $H_2SO_4$ (0.25 mL), EtOAc (10 mL) was added, and the whole was stirred vigorously, precipitating the enzyme. The solution was filtered through a sintered glass filter with a silica bed, and washed with EtOAc (10 mL). The aqueous layer was extracted with EtOAc (2 ×10 mL) and the combined

organic layer was washed with brine (10 mL). The organic layer was dried over $Na_2SO_4$ and filtered, and the solvent was removed under reduced pressure to afford the residue. Product formation was analyzed through TLC and $^1$H NMR spectra recorded in acetone-$d_6$.

**Enzymatic reduction of 2-tetralone (14)**

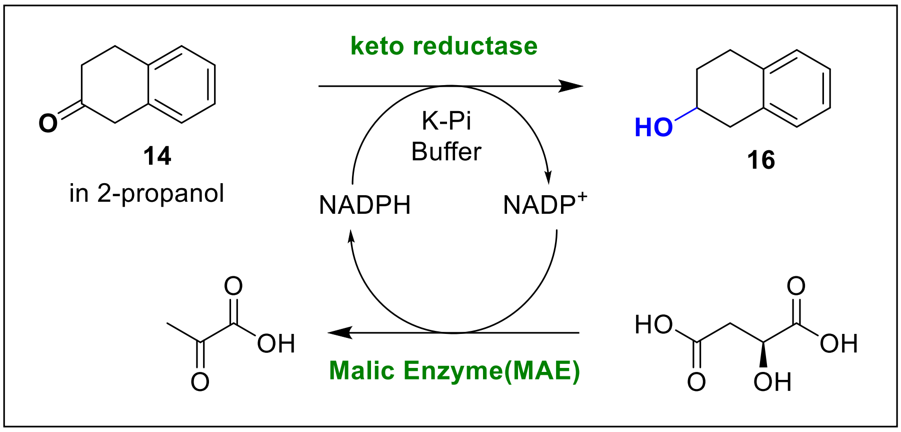


**Figure S2.** Reduction of 2-tetralone (**14**) with KR1_his, KR2_his or ActIII KR_his.

To a buffer solution (10.0 mL, 50 mM KPi, 10 µM $MnCl_2$, pH 7.0), current of argon was bubbled for half an hour. Then, L-malic acid (92.0 mg, 0.68 mmol, 5.0 equiv), NADP-Na (10.7 mg, 0.013 mmol, 0.1 equiv., 10%) and MAE (500 µL, 40 U/mL) were added and the mixture was stirred slowly under argon at room temperature. After 1h, the substrate, 2-tetralone (**14**) (20.0 mg, 0.136 mmol, 1 equiv.) in 2-propanol (0.5 mL, 5% v/v) was added slowly, while stirring at 100 rpm. At last, the enzyme (KR1_his, KR2_his and ActIII KR_his) (2 mL, 3.3 U/mL) was added and the mixture was stirred at room temperature. After 24 h, the solution was acidified with 10% of $H_2SO_4$ (0.25 mL) and EtOAc (10 mL) was added and stirred vigorously, precipitating the enzyme. The solution was filtered with sintered glass with silica bed and washed with EtOAc (10 mL). The aqueous layer was extracted (x2) with EtOAc and the combined organic layer was washed with brine (10 mL). The organic layer was dried over $Na_2SO_4$, filtered and the solvent was removed under reduced pressure to afford a dark brown residue **16**, which was purified by using column chromatography.

**$R_f$** = 0.35 (cyclohexane/ethyl acetate = 3:2)

**Yield:** 17 mg, 85%.

**$^1$H NMR (400 MHz, $CDCl_3$):** δ (ppm) 7.14–7.06 (m, 4H), 4.20–4.11 (m, 1H), 3.12–3.06 (dd, $^2J$ = 15.0 Hz, $^3J$ = 3.0 Hz,1H), 3.01–2.84 (m, 2H), 2.81–2.73 (dd, $^2J$ = 18.0 Hz, $^3J$ = 6.0, 1H), 2.11–2.01 (m, 1H), 1.88–1.76 (m, 1H), 1.71 (1H, br s,).[7]

**$C_{10}H_{12}O$:** 148.09 g.mol$^{-1}$

| Substrate | Enzymes | Product | Conversion[a] ($^1$H NMR) % |
|---|---|---|---|
| 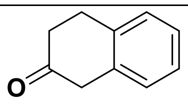 **14** | KR1 | 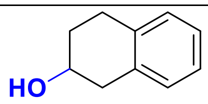 **16** | 100 |
| | KR2 | | 13 |
| | Act III | | 30 |

[a]Conversion was measured using $^1$H NMR spectroscopy.

## Enzymatic reduction of 1-tetralone (15)

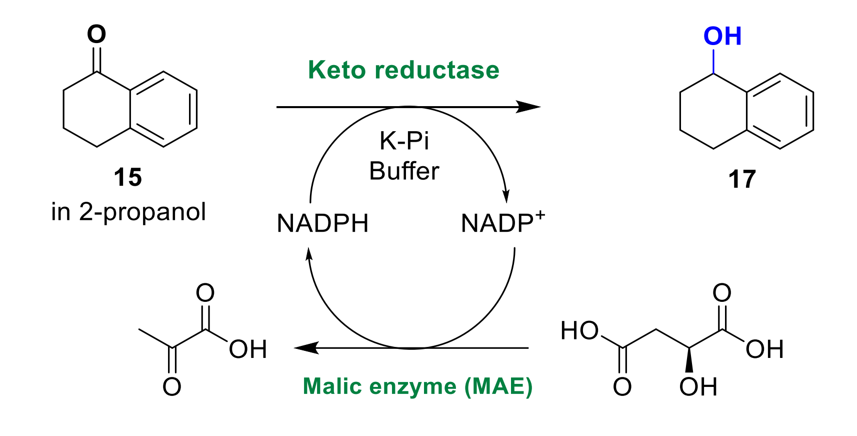


**Figure S3.** Reduction of 1-tetralone (**15**) with KR1_his, KR2_his or ActIII KR_his.

To a buffer solution (10.0 mL, KPi = 50 mM, 10 µM $MnCl_2$, pH 7.0), current of argon was bubbled for half an hour. Then, L-malic acid (92.0 mg, 0.68 mmol, 5.0 equiv), NADP-Na (10.7 mg, 0.013 mmol, 0.1 equiv., 10%) and MAE (500 µL, 40 U/mL) were added and the mixture was stirred slowly under argon at room temperature. After 1h, the substrate, 1-tetralone (**15**) (20.0 mg, 0.136 mmol, 1 equiv.) in 2-propanol (0.5 mL, 5% v/v) was added slowly, while stirring at 100 rpm. At last, the enzyme (KR1_his, KR2_his and ActIII KR_his) (2 mL, 3.3 U/mL) was added and the mixture was stirred at room temperature. After 24 h, the solution

was acidified with 10% of $H_2SO_4$ (0.25 mL) and EtOAc (10 mL) was added and stirred vigorously, precipitating the enzyme. The solution was filtered with sintered glass with silica bed and washed with EtOAc (10 mL). The aqueous layer was extracted (x2) with EtOAc and the combined organic layer was washed with brine (10 mL). The organic layer was dried over $Na_2SO_4$, filtered and the solvent was removed under reduced pressure to afford a dark brown residue **17**, which was purified by using column chromatography.

**$R_f$** = 0.30 (hexane/ethyl acetate 80:20)

**Conversion:** 20% (assigned by $^{1}$H NMR spectroscopy)

**Yield:** 3 mg, 15%.

**$^{1}$H NMR (400 MHz, dmso-*$d_6$*):** δ (ppm) dppm 7.38 (m, 1H), 7.13 (m, 2H), 7.04 (m, 1H), 5.08 (d, 1H, $^{3}J$ = 6.0 Hz), 4.55 (m, 1H), 2.59–2.78 (m, 2H), 1.82–1.94 (m, 2H), 1.59–1.73 (m, 2H).[8]

**$C_{10}H_{12}O$:** 148.09 g.mol$^{-1}$

| Substrate | Enzymes | Product | Conversion[a] ($^{1}$H NMR) % |
|---|---|---|---|
| 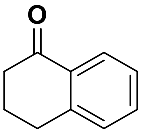 **15** | KR1 | 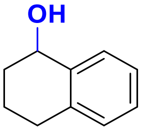 **17** | 20 |
| | KR2 | | <2 |
| | Act III | | <5 |

[a]Conversion was measured using $^{1}$H NMR spectroscopy.

## VI. References


1. Husain, S. M.; Schätzle, M. A.; Röhr, C.; Lüdeke, S.; Müller, M. *Org. Lett*. **2012**, *14*, 3600–3603.
2. Dachavaram, S. S.; Kalyankar, K. B.; Das, S. *Tetrahedron Lett*. **2014**, *55*, 5629–5631.
3. Tsujihara, K.; Hongu, M.; Saito, K.; Kawanishi, H.; Kuriyama, K.; Matsumoto, M.; Oku, A.; Ueta, K.; Tsuda, M.; Saito, A. *J. Med. Chem*. **1999**, *42*, 5311–5324.
4. Schätzle, M. A.; Flemming, S.; Husain, S. M.; Richter, M.; Günther, S.; Müller. M. *Angew. Chem. Int. Ed*. **2012**, 51, 2643–2646.
5. Saha, N.; Müller, M.; Husain, S. M. *Org. Lett.* **2019**, 21, 2204–2208.
6. Schätzle, M. A.; Husain, S. M.; Ferlaino, S.; Müller, M. *J. Am. Chem. Soc.* **2012**, *134*, 14742–14745.
7. Soni, R.; Collinson, J.; Clarkson, G.C.; Wills, M. *Org. Lett.*, **2011**, *13*, 4304–4307.
8. Bichlmaier, I.; Siiskonen, A.; Kurkela, M.; Finel, M.; Yli-Kauhaluoma, J. *Biol. Chem.* **2006**, *387*, 407–416.
